# A new silver coordination polymer based on 4,6-diamino-2-pyrimidinethiol: synthesis, characterization and catalytic application in asymmetric Hantzsch synthesis of polyhydroquinolines

**DOI:** 10.1038/s41598-021-94846-6

**Published:** 2021-08-02

**Authors:** Noorullah Hussain-Khil, Arash Ghorbani-Choghamarani, Masoud Mohammadi

**Affiliations:** 1 Department of Chemistry, Faculty of Science, Ilam University, Ilam, Iran; 2 Department of Organic Chemistry, Bu-Ali Sina University, Hamedan, 6517838683 Iran

**Keywords:** Catalysis, Coordination chemistry, Green chemistry, Organic chemistry

## Abstract

A highly efficient and stable heterogeneous coordination polymer (CP) was successfully prepared by hydrothermal combination of silver and 4,6-diamino-2-pyrimidinethiol. The prepared coordination polymer was characterized by FT-IR, XRD, TGA, SEM, EDX, X-ray mapping and Nitrogen adsorption–desorption analysis. The prepared Ag–CP exhibit excellent catalytic activity in multicomponent Hantzsch synthesis of polyhydroquinolines under mild reaction conditions in relatively short reaction times. The heterogeneity of the catalyst was confirmed by the hot filtration test; also, the catalyst was reused for at least four times under the optimized reaction conditions without any significant loss of its catalytic activity.

## Introduction

Several important applications of organosilver compounds in organic chemistry have been recognized for a long time, i.e. applying silver reagents in the several reactions such as cycloaddition reactions, cyclization, coupling reactions and C-H functionalization, carbene, nitrene, silylene-transfer reactions and so on^1–6^. From the environmental and economic points of view, catalytic functional groups transformation technologies signify an important tool for the development of “green chemistry”, which means less waste generation and little energy consumption, as well as high atom economy and environmental friendliness^7, 8^. Accordingly, as a result of their high catalytic performance, many transition metals, especially valuable metals such as ruthenium, platinum, palladium and iridium, are applied in these transformations^9–12^. However, in view of their limited abundance on earth, high prices and toxicity, chemists began to scrutinize new catalyst systems using first and second-row transition metals^13^. Currently, cheap, abundant and low-toxic metals—such as silver—have gained widespread attention of the synthetic community. In this sense, the coordination polymers have caught the attention as catalytic systems for many heterogeneous industrial reactions^14–19^. These types of heterogeneous catalysts are attractive due to their structural ordering, large size and volume of pores, and large specific surface area^20–23^. Furthermore, when compared to conventional mesostructured silica-based supports, the CPs ones have shown much higher activity and selectivity by providing good and regular interaction between metal species and organic reagents^24, 25^. It has been found out that Ag-based CPs show high surface area and, as a result, a large number of reactions happen in presence of Ag–CP as catalyst^26–29^. Actually, in a number of cases, such reactions occur more proficiently and with more selectivity, as compared to the reactions carried out in presence of other types of heterogeneous nanomaterials. Such reactions are simple to handle, can diminish pollution, are comparatively cheaper in industrial sector.

Moreover, multicomponent reactions (MCRs) are synthetic protocols which are able to join three or more substrates together in a highly regio- and stereoselective manner in order to deliver the structurally complex organic molecules^30–32^. Accordingly, it is worth mentioning that they have been remarkably applied in all fields of organic synthesis^7, 13, 33–36^. But, regarding the one pot synthesis, they give yield in a highly stereoselective manner which is useful for the organic transformations^37–39^. MCRs have remarkable benefits in terms of simplicity and synthetic efficiency over formal chemical reactions and show high atom economy and high selectivity^40–43^. Therefore, the use of MCRs as well as domino reaction sequences has significantly increased for a large number of products^44^. Nowadays, we are fascinated in developing a facile MCR procedure to synthesize polyhydroquinolines, in view of their interesting applications in medicinal and materials chemistry. The synthesis of polyhydroquinoline derivatives can be regarded as an example of the Hantzsch dihydropyridine (Pyridine) synthetic method^13^. Polyhydroquinolines have fascinated much interest because of their diverse pharmacological and therapeutic properties^13^. Most of the reported methods to synthesize polyhydroquinolines possess some specific drawbacks such as low yield, harsh reaction condition and use of volatile organic solvent^7, 45–47^. The use of a variety of homogeneous and heterogeneous catalysts has been previously reported to synthesize various densely substituted polyhydroquinolines via traditional organic synthesis or MCR synthesis^37, 48–53^. However, we have developed a new protocol with environmentally benign condition and also with the high catalytic efficiency of the novel Ag–CP by the condensation reaction of the substituted aromatic aldehydes, ethyl acetoacetate, dimedone and ammonium acetate with excellent yields. Moreover, this catalyst can be easily separated from the reaction solution and would also exhibit an endurable catalytic activity in the long-term reaction.

## Experimental

### Preparation of Ag–CP

**Scheme 1:**
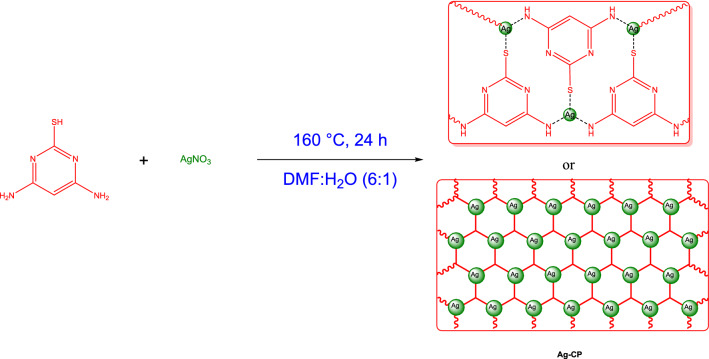
The synthesis of Ag–CP.

A solution of 4,6-diamino-2-pyrimidinethiol (1 mmol) in water (2 mL) was prepared and, then, added to a solution of AgNO_3_ (2 mmol) in DMF (12 mL). The obtained mixture was stirred under darkness at 80 °C for 20 min. Afterwards, the mixture was kept in an autoclave at 160 °C for 24 h (Scheme1). In the next step, the obtained powder from the autoclave was cooled down and washed with ethyl acetate. Subsequently, the obtained Ag–CP black powder was sonicated for 20 min, dried in room temperature and, finally, stored in a dark brown bottle.

### General procedure for the catalytic synthesis of polyhydroquinolines

A mixture of aromatic aldehydes (1.0 mmol), ethyl acetoacetate (1 mmol), dimedone (1 mmol), NH_4_OAc (1.2 mmol) and Ag–CP (8 mg) was stirred in EtOH under reflux conditions for the required time. The progress of reaction was monitored by TLC. After completion of the reaction, the mixture was cooled down to room temperature. Afterwards, the catalyst was separated using simple filtration and, then, washed by hot ethyl acetate. Finally, the solvent was evaporated and the pure polyhydroquinoline products was obtained through recrystallization in ethanol.

### Selected spectral data

#### Ethyl-4-(4-methylphenyl)-2,7,7-trimethyl-5-oxo-1,4,5,6,7,8-hexahydroquinoline-3-carboxylate

^1^H NMR (500 MHz, DMSO): δ (ppm) 0.84 (s, 3H), 1.00 (s, 3H), 1.12 (t, J = 7.2 Hz, 3H), 1.95 (d, J = 16.0 Hz, 1H), 2.14–2.19 (m, 4H), 2.23–2.31 (m, 4H), 2.41 (d, J = 16.8 Hz, 1H), 3.96 (q, J = 7.2 Hz, 2H), 4.80 (s, 1H), 6.99 (d, J = 7.6 Hz, 2H), 7.02 (d, J = 7.6 Hz, 2H), 9.01 (s, 1H); ^13^C NMR (126 MHz, DMSO) δ 194.29, 166.92, 149.40, 144.83, 144.80, 134.57, 128.34, 127.41, 110.11, 103.79, 59.04, 35.42, 32.17, 29.12, 27.05, 20.83, 18.28, 14.20.

#### Ethyl-4-(3-hydroxyphenyl)-2,7,7-trimethyl-5-oxo-1,4,5,6,7,8-hexahydroquinoline-3-carboxylate

^1^H NMR (500 MHz, DMSO): δ (ppm) 0.87 (s, 3H), 1.00 (s, 3H), 1.16 (t, J = 7.2 Hz, 3H), 1.97 (d, J = 16.0 Hz, 1H), 2.17 (d, J = 16.0 Hz, 1H), 2.27 (m, 4H), 2.40 (d, 1H), 4.00 (m, 2H), 4.79 (s, 1H), 6.47 (d, J = 7.2 Hz, 1H), 6.58–6.63 (m, 2H), 6.90–6.96 (m, 1H), 9.01 (s, 1H), 9.07 (s, 1H); ^13^C NMR (126 MHz, DMSO) δ 194.31, 166.98, 156.86, 149.48, 149.00, 144.76, 128.56, 118.11, 114.69, 114.50, 109.94, 103.65, 59.06, 49.87, 35.64, 32.17, 29.18, 26.58, 18.35, 14.21.

#### Ethyl-4-(4-chlorophenyl)-2,7,7-trimethyl-5-oxo-1,4,5,6,7,8-hexahydroquinoline-3-carboxylate

1H NMR (400 MHz, DMSOd6): δ (ppm) 0.82 (s, 3H), 0.99 (s, 3H), 1.10 (t, J = 7.2 Hz, 3H), 1.98 (d, J = 15.6 Hz, 1H), 2.18 (d, J = 14.4 Hz, 1H), 2.26—2.29 (m, 4H), 2.41 (d, J = 17.2 Hz, 1H), 3.96 (q, J = 6.8 Hz, 2H), 4.84 (s, 1H), 7.11–7.19 (m, 2H), 7.21–7.29 (m, 2H), 9.11 (s, 1H); ^13^C NMR (126 MHz, DMSO) δ 194.29, 166.68, 161.23, 149.65, 146.61, 145.47, 130.23, 129.36, 127.72, 109.70, 103.14, 59.14, 50.17, 35.67, 35.60, 32.16, 29.12, 26.45, 18.38, 18.31, 14.15.

## Results and discussion

### Structural and chemical composition analysis

**Figure 1:**
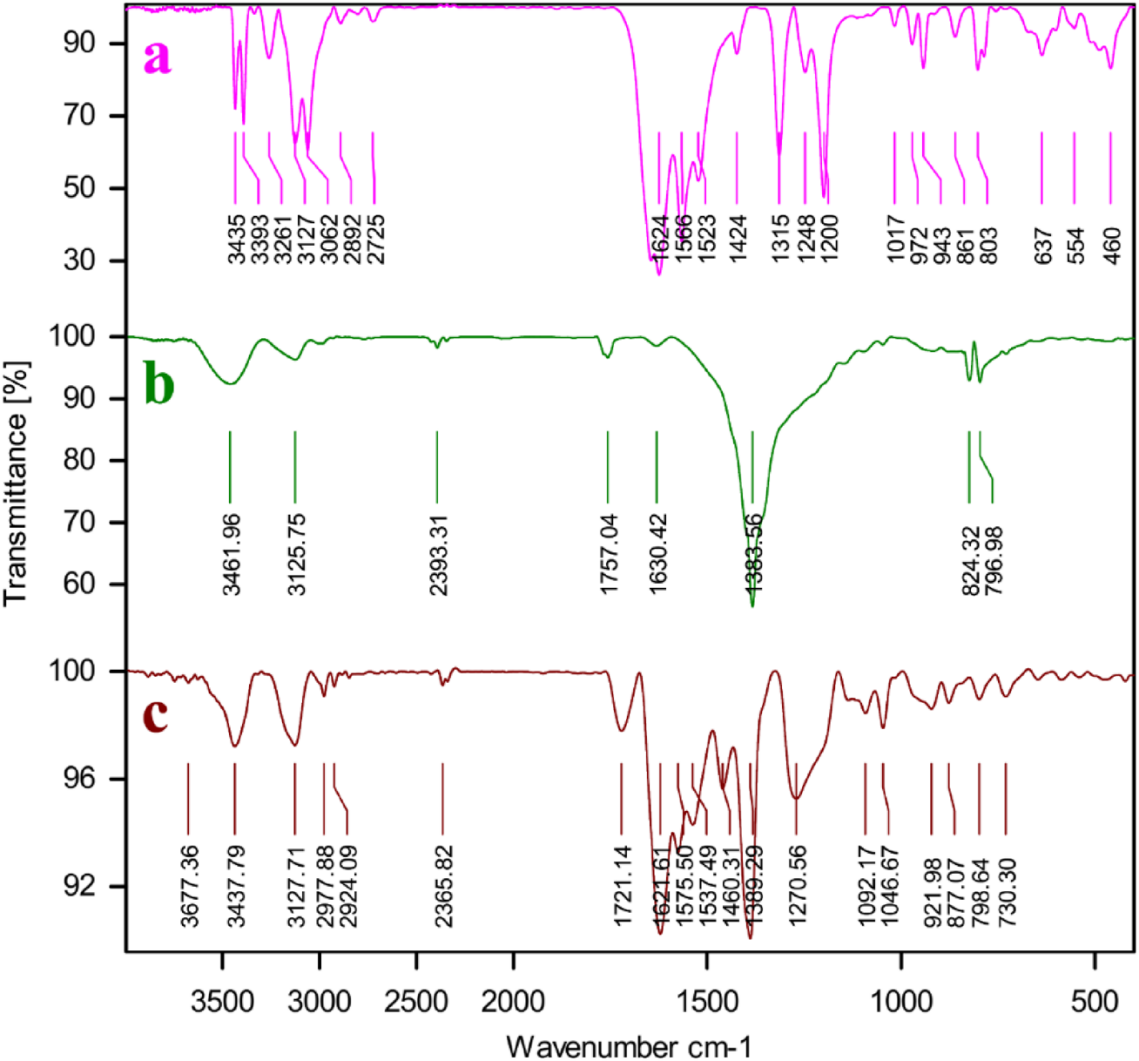
FT-IR Spectrums of (**a**) 4,6-diamino-2-pyrimidinethiol, (**b**) Siler nitrate and (**c**) Ag–CP.

The FT-IR spectrum of the 4,6-Diamino-2-pyrimidinethiol (Fig. 1a) before of the CPs complexion had absorption bands in the regions of 3339 cm^−1^ and 3435 cm^−1^ respectively, related to the stretching vibration of the N–H bonds of free NH_2_ groups. This bands disappeared in the FT-IR spectra of the Ag–CP (Fig. 1c), which is indicative of the fact that the NH_2_ groups of 4,6-diamino-2-pyrimidinethiol have been deprotonated and coordinated to the Ag atoms. Moreover, a shift on the bending vibration of NH_2_ near 1628 cm^−1^ in the Ag–CP to more higher wavenumbers in comparison to the 4,6-Diamino-2-pyrimidinethiol demonstrates the existence of the metal coordination bonding and confirms the successful complexion of Ag ions with the nitrogen atoms and thiol atoms of ligand. In the curve of the Ag–CP (Fig. 1c), the strong C=C stretching vibration band at 1460 cm^−1^, and C–N stretch band at 1092 cm^−1^ provide evidences confirming the successful synthesis of Ag–CP. On the basis of the FT-IR, we can also observe that the FT-IR spectrum of Ag–CP obtained from silver nitrate (Fig. 1b) shows sharp characteristic peaks suggesting the high crystalline nature of Ag–CP. On the basis of the FT-IR results Silver ions were coordinated to the amine and thiol functional groups of 4,6-Diamino-2-pyrimidinethiol as a tridentate ligand and confirm the suggested structure in Scheme 1.

**Figure 2:**
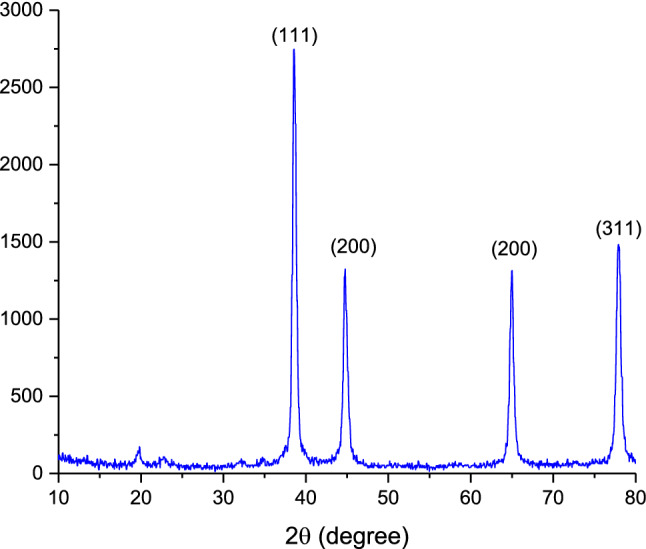
PXRD pattern of Ag–CP.

The synthesized CPS materials were characterized by PXRD analysis using PW1730 instrument from Philips Company having CuKα (λ = 1.540598 Å) radiation at 40 kV and 30 mA with 2θ = 10°–80°. The XRD pattern of Ag–CP is shown in Fig. 2. According to powder PXRD standards (PXRD, Ref. No. 01-087-0718), the crystalline peaks occurring at 2θ = 38.48°, 44.77°, 64.99°and 77.92° can be attributed to the (111), (200), (220), and (311) crystallographic planes of silver crystals, which are in agreement with the previously reported literatures^54^. The PXRD patterns shown in Fig. 2 confirm the successful coordination of silver ions within the prepared framework. In addition, the (111) Ag diffraction peak with appreciable intensity further confirms the presence of Ag metal in the prepared Ag–CP^55^.

**Figure 3:**
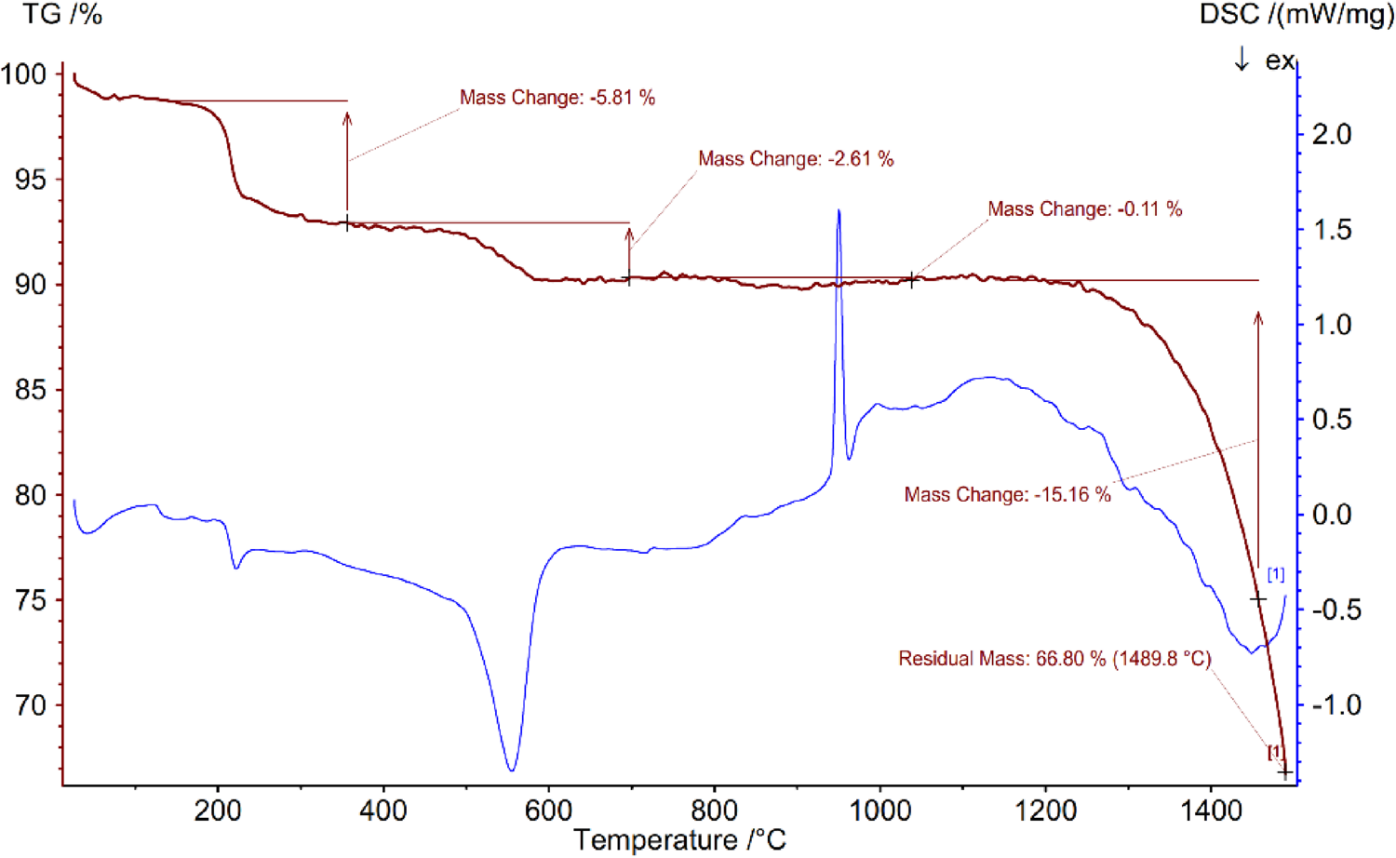
TGA/DSC curves of Ag–CP.

The mass ratios and the thermal stability of Ag–CP were examined by the thermogravimetric analysis (TGA) (Fig. 3). In all of the TGA curve, the weight loss which was occurred below 200 °C was attributed to the release of physically adsorbed moisture and water and organic solvents from the sample^56^. It was at above 200 °C that the framework degradation started. The main weight loss at 250–600 °C was caused by the decomposition of 4,6-Diamino-2-pyrimidinethiol ligand^57, 58^. This result confirms the successful synthesis of Ag–CP, and the fact that the temperature stability of the sample is about 200 °C. The DSC results which support the TGA data, based on weight loss of the sample, approve the range of temperature stability of the sample.

**Figure 4:**
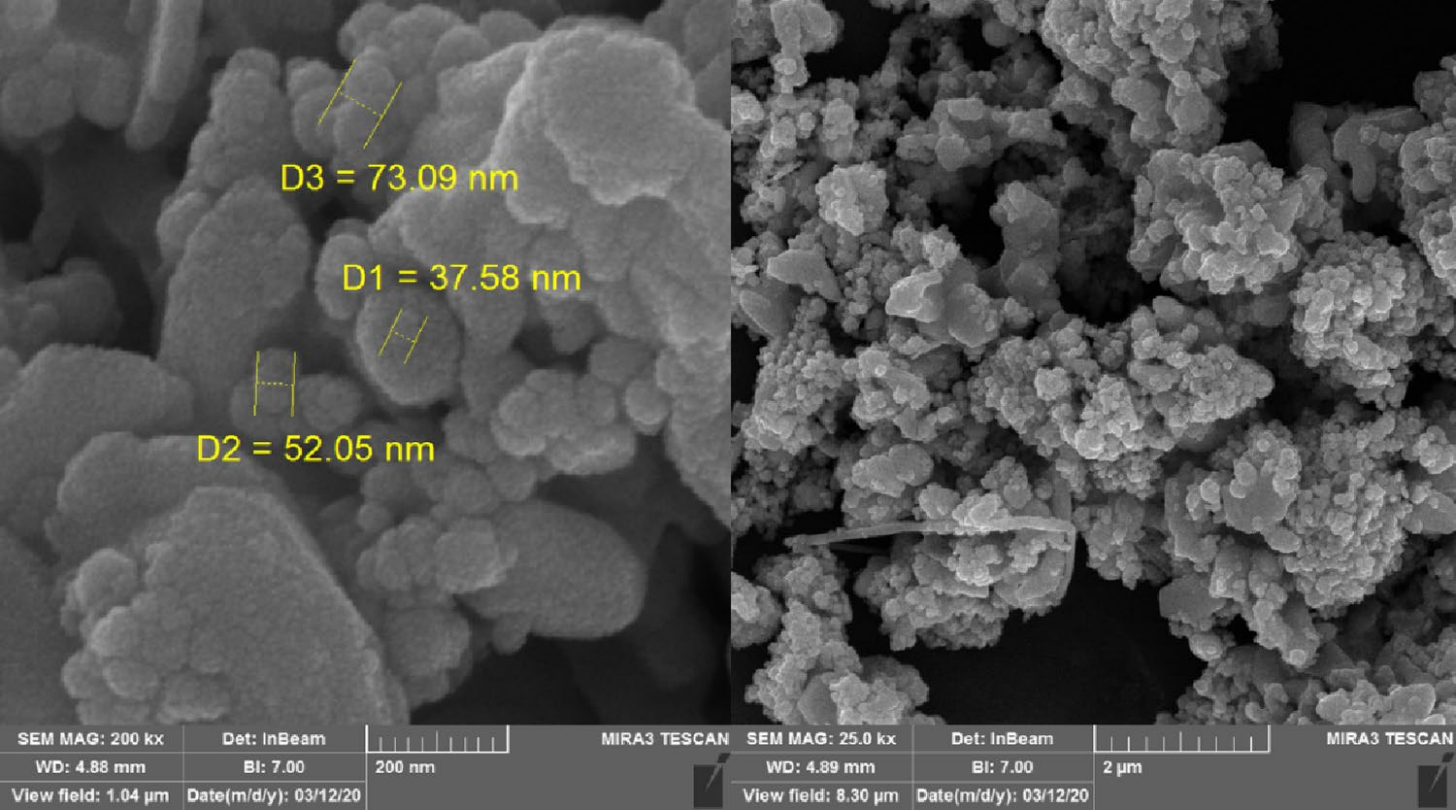
SEM images of Ag–CP.

As shown in the Fig. 4, the morphology of the surfaces of the obtained Ag–CP porous material was further characterized by scanning electron microscopy. A closer look at the SEM images of the obtained porous material indicates that the Ag–CP have been formed in uniform nanometer‐sized particles.

**Figure 5:**
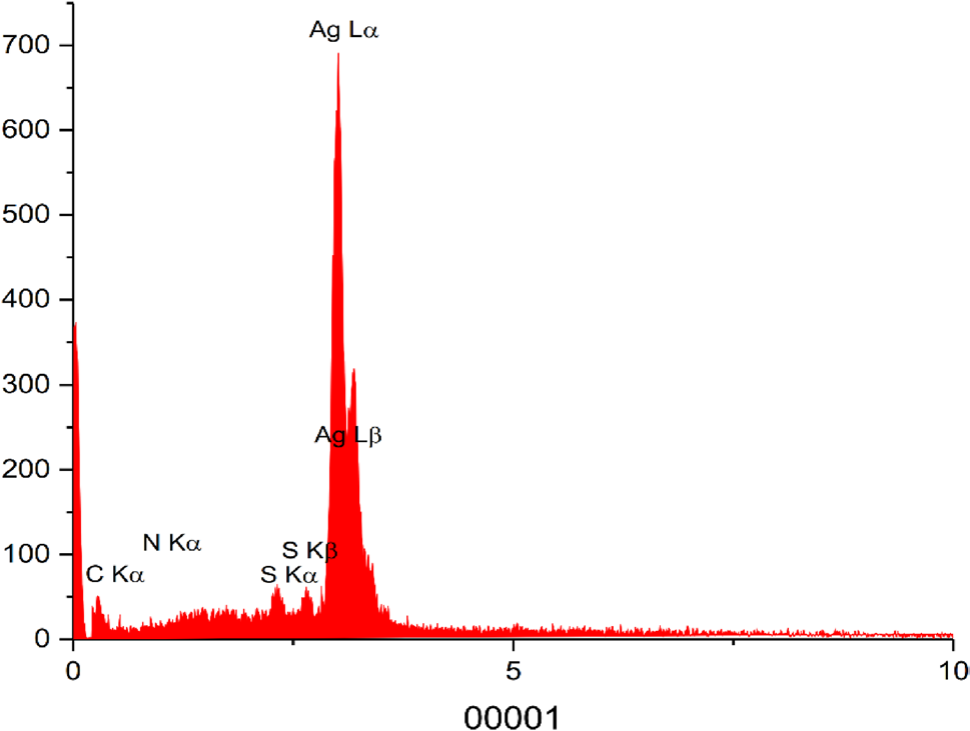
EDX Analysis of Ag–CP.

The EDX spectrum showed that a lot of Ag species was observed on the surface of the obtained Ag–CP porous catalyst in Fig. 5. The presence of C, S and N elements in the Ag–CP was also confirmed by energy dispersive X-ray (EDX) measurements.

**Figure 6:**
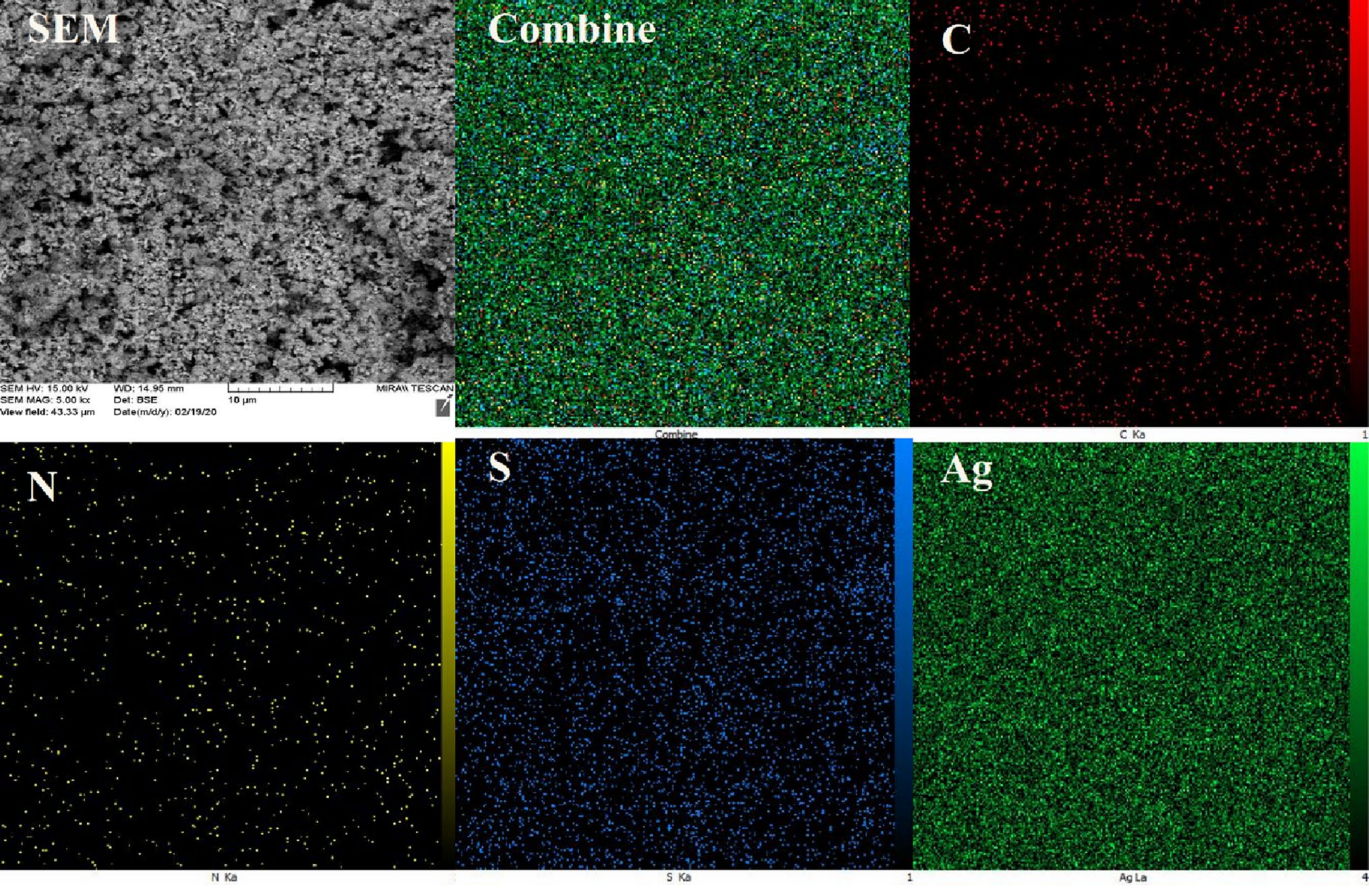
X-ray mapping Analysis of Ag–CP.

The X-ray mapping technique was performed in order to complete the characterization of the Ag–CP elements and how they are distributed in the CPs structure. Figure 4 shows the elemental X-ray mapping of Ag–CP. The uniform distribution of the index elements (C, S, N, and Ag) is observed at the obtained porous material. The uniform distribution of C, N and S elements indicated the presence of the 4,6-diamino-2-pyrimidinethiol scaffold, which has been used as a ligand. In addition, the uniform distribution of the Ag element can be seen in the X-ray-mapping images. It can be concluded from the figure that the Ag has evenly coordinated to 4,6-diamino-2-pyrimidinethiol scaffold, and a good catalytic surface has been formed via the uniform incorporation of Ag catalytic species with nitrogen and sulfur groups (Fig. 6).

**Figure 7:**
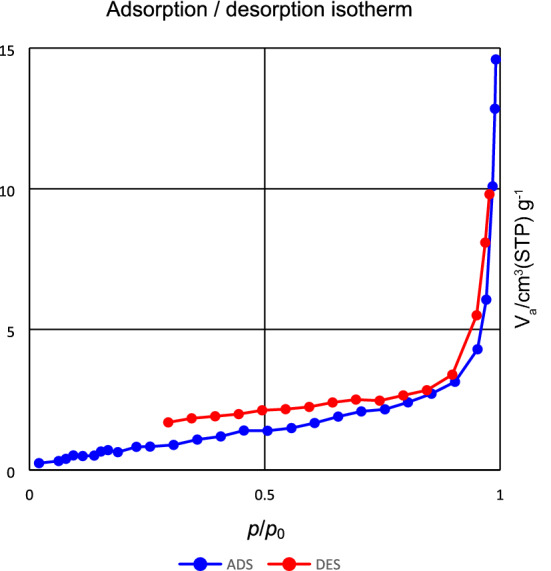
N_2_ adsorption/desorption isotherms of the Ag–CP.

The surface area and pore size distribution of Ag–CP are studied by BET analysis which exhibits type IV adsorption isotherm according to IUPAC classification. The appeared characteristic hysteresis loop is in good agreement with CPs material (Fig. 7). Regarding the BET analysis, the calculated surface area of Ag–CP is 3.59 m^2^/g. The pore volumes and pore size distribution of Ag–CP are calculated by BJH analysis and the values are 0.02 cm^3^ g^-1^, and 23.45 nm, respectively. Which indicated that the obtained Ag–CP is a mesoporous material. These results are in agreement with the reported coordination compounds in literature^59, 60^.

### Catalytic study

After the successful characterization of the synthezed Ag–CP, its catalytic activity was evaluated in multicomponent Hantzsch condensation of polyhydroquinolines under diverse conditions (Table 1). Firstly, the Hantzsch reaction was carried out between *para*-chlorobenzaldehyde and dimedone, ethyl acetoacetate and ammonium acetate as a model reaction. Afterwards, we aimed at investigating the optimum reaction conditions in presence of the newly synthesized Ag–CP catalyst. Table 1 clearly depicts that the reaction progress is highly affected with catalyst loading, solvent and temperature. At the first step, the effect of Ag–CP loading to catalyze the reaction was examined by varying the amount of Ag–CP in the model reaction. It was observed that the yield of the polyhydroquinoline product enhanced with increasing the amount of the catalyst from 1 to 7 mg (Table 1, entries 4–10). The best result in an appropriate time was obtained using 7 mg of the catalyst (Table 1, entry 10). Subsequently, the effect of different solvents with varying polarity (DMSO, DMF, PEG-400, EtOH and EtOH:H_2_O (1:1)) was studied (Table 1, entries 10 and 12–15) and the best reaction yield was carried out in EtOH as solvent. With increasing the temperature from room temperature to 80 °C, a dominant increase in the yield was observed (Table 1, entries 10 and 16–18). Regarding the optimization studies, the optimum conditions for this reaction are: Ag–CP (7 mg) in the EtOH at reflux conditions (Table 1, entry 10). Additionally, the catalytic effect of 4,6-diamino-2-pyrimidinethiol and Ag(NO_3_)_2_ was investigated on the model reaction. It was observed that 4,6-diamino-2-pyrimidinethiol and Ag(NO_3_)_2_ cannot efficiently catalyze the reaction and, as a result, the product is obtained in low yields in 85 min.

**Table 1:** Optimization of the reaction conditions for the Hantzsch condensation of *para*-Chlorobenzaldehyde, dimedone, ethyl acetoacetate and ammonium acetate as a model reaction for the synthesis of polyhydroquinolines.

	
Entry	Catalyst	Amount of catalyst (mg)	Solvent	Temperature (°C)	Time (min)	Yield (%)^a,b^
1	–	–	EtOH	Reflux	85	17
2	4,6-diamino-2-pyrimidinethiol	7	EtOH	Reflux	85	23
3	Ag(NO_3_)_2_	7	EtOH	Reflux	85	Trace
4	Ag–CP	4	EtOH	Reflux	85	69
5	Ag–CP	5	EtOH	Reflux	85	74
6	Ag–CP	6	EtOH	Reflux	85	78
7	Ag–CP	7	EtOH	Reflux	85	87
8	Ag–CP	7	EtOH:H_2_O	80	90	82
9	Ag–CP	7	PEG-400	80	120	75
10	Ag–CP	7	DMF	80	110	74
11	Ag–CP	7	DMSO	80	125	78
12	Ag–CP	7	EtOH	25	8 h	NR
13	Ag–CP	7	EtOH	60	70	76
14	Ag–CP	7	EtOH	70	75	79

^a^Isolated yield.

^b^Reaction conditions: 4-Chlorobenzaldehyde (1 mmol), dimedone (1 mmol), ethyl acetoacetate (1 mmol), ammonium acetate (1.2 mmol), catalyst (mg) and solvent (3 mL).

After optimization of the reaction conditions, we have explored the scope of the reaction with various electron-donating and electron-withdrawing groups of aldehydes (Table 2). Both these substituents gave excellent yield of the product.

**Table 2:** Hantzsch synthesis of polyhydroquinoline derivatives in the presence of Ag–CP in EtOH at 80 °C.


Entry	Aryl aldehyde	Product	Time (min)	Yield (%)^a,b^	Melting point	
					Measured	Literature
1			80	93	198–205	203–206^61^
2			85	87	245–248	246–247^61^
3			80	86	245–249	249–251^61^
4			86	82	249–251	252–255^47^
5			80	83	254–256	255–257^47^
6			85	87	197–202	204–206^61^
7			85	88	264–268	246–248^62^
8			80	93	169–174	175–176^61^
9			80	87	208–210	230–232^37^
10			85	81	238–240	231–233^47^
11			85	74	294–295	305–307^63^
12			90	78^c^	298^d^	298–300^64^

^a^Isolated yields.

^b^Reaction conditions: Aromatic aldehyde (1 mmol), dimedone (1 mmol), ethyl acetoacetate (1 mmol), ammonium acetate (1.2 mmol), Ag–CP (7 mg) and EtOH (3 mL) at 80 °C reflux conditions.

^c^Reaction conditions: Aromatic aldehyde (1 mmol), dimedone (2 mmol), ethyl acetoacetate (2 mmol), ammonium acetate (2.4 mmol), Ag–CP (14 mg) and EtOH (6 mL) at 80 °C reflux conditions.

^d^Decomposition.

**Scheme 2:**
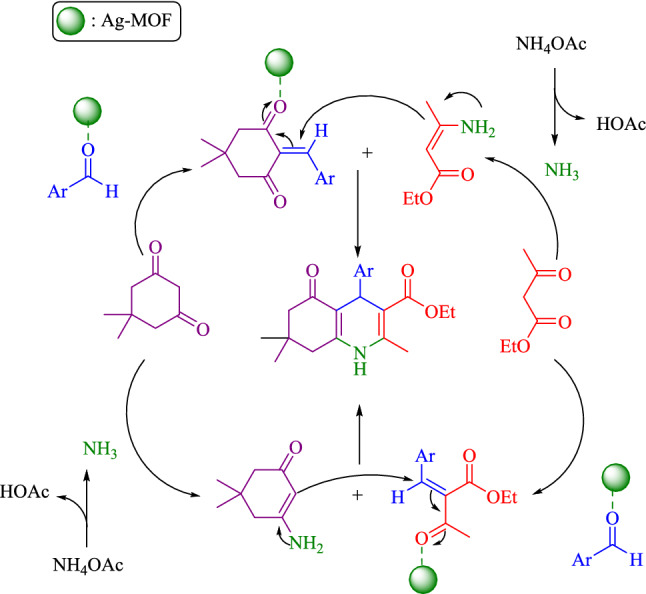
Proposed mechanism for the synthesis of polyhydroquinolines in the presence of Ag–CP.

A plausible reaction mechanism for the Hantzsch synthesis of polyhydroquinoline derivatives in the presence of Ag–CP is depicted in Scheme 2. Initially, the carbanion was formed by proton abstraction from the active methylene compounds (ethyl acetoacetate or dimedone) which underwent Knoevenagel condensation reaction with aldehyde to form an α,β-unsaturated compound. In the second part of the reaction, ammonium acetate gave acetic acid and ammonia and, then, the ammonia combined with the active carbonyl compounds (ethyl acetoacetate or dimedone) and attained imine derivatives. Finally, the Michael addition of imine derivatives on the α,β-unsaturated carbonyl compounds (which were activated by Ag–CP) was followed by cyclization reaction. Besides, dehydration gave the final polyhydroquinoline products (Scheme 2).

### Hot filtration

The hot filtration test was another analysis to approve the heterogeneous nature of the Ag–CP in the Hantzsch synthesis of polyhydroquinolines. On this basis, the model reaction was studied again under the optimized reaction condition. After 43 min (59% conversion), the Ag–CP were removed from the reaction by simple filtration. Afterwards, the rest of the reaction was stirred in the absence of the catalyst for a further 43 min. The obtained results show that the Ag-based framework played a catalytic role in the reaction without the Ag leaching into the solution or framework degradation.

### Recyclability study

**Figure 8:**
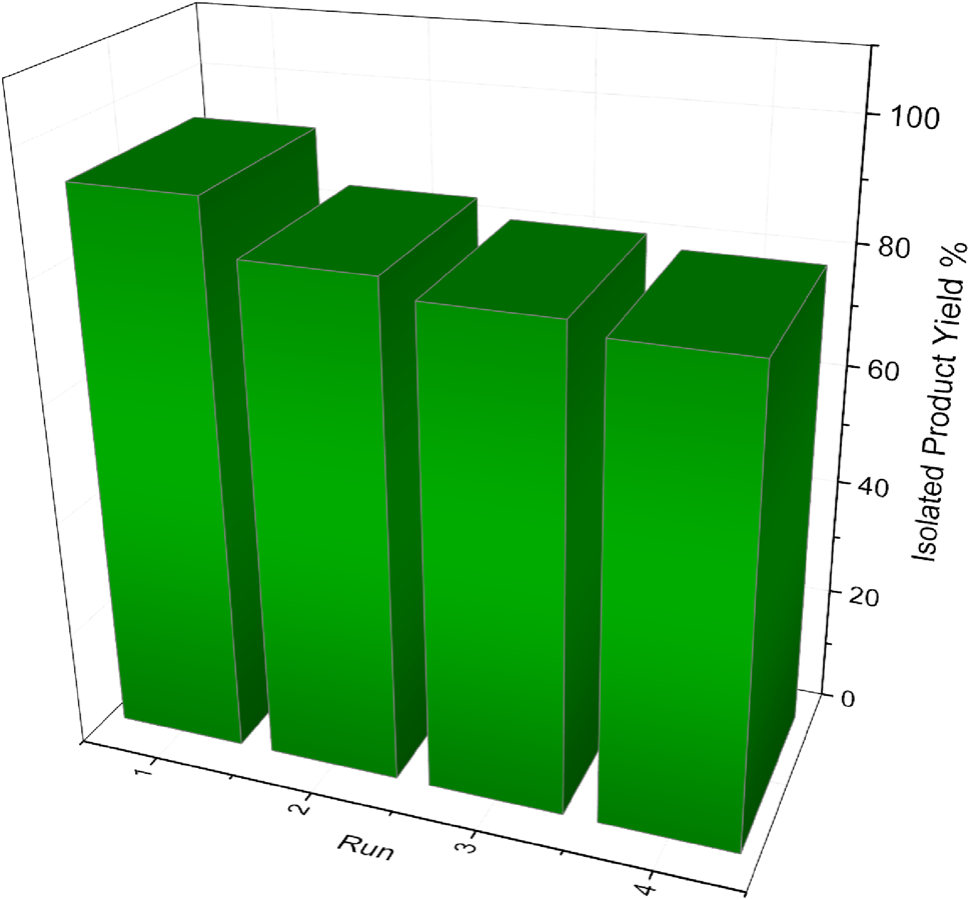
Recyclability of the Ag–CP.

Considering the environmental and economic factors, and also the principles of green chemistry, the long-term durability experiment of the prepared catalyst has been investigated. In this sense, the model reaction was carried out under the optimized reaction conditions to test the reusability behaviour of the Ag–CP (Fig. 8). Briefly, after the finalization of each run of the Hantzsch reaction, which was monitored by the thin-layer chromatography, the reaction mixture was cooled to room temperature and, then, the heterogeneous catalyst was separated by simple filtration, washed with Acetone, EtOAc and H_2_O and, finally, dried in an oven at 60 °C overnight to be used in the next run. As shown in Fig. 8, no significant decrease in the yield of the reactions was observed even after four runs. These results confirmed the high catalytic activity and long-term durability of the Ag–CP in the Hantzsch synthesis of polyhydroquinolines.

**Figure 9:**
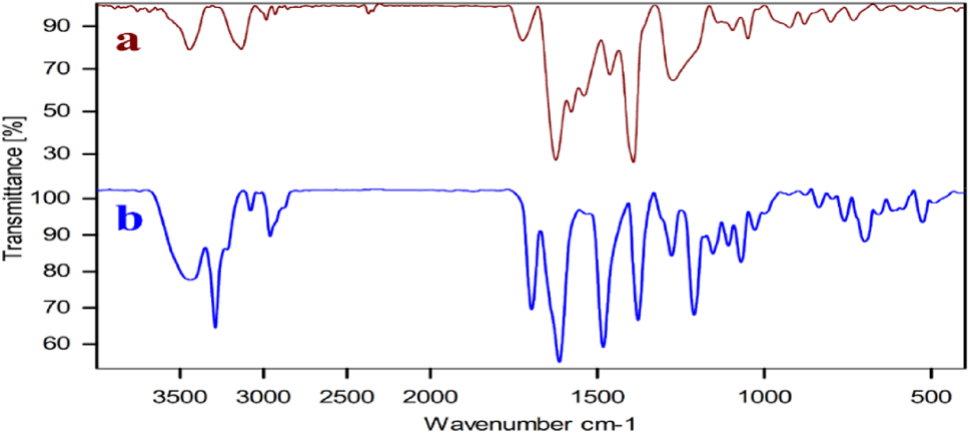
FT-IR spectra of the (**a**) fresh Ag–CP and (**b**) spent Ag–CP.

The Ag–CP have been characterized by FT-IR before and after the reaction (Fig. 9a, b). These spectra showed the similar peaks and indicating the stability of this catalyst under applied reaction conditions.

**Figure 10:**
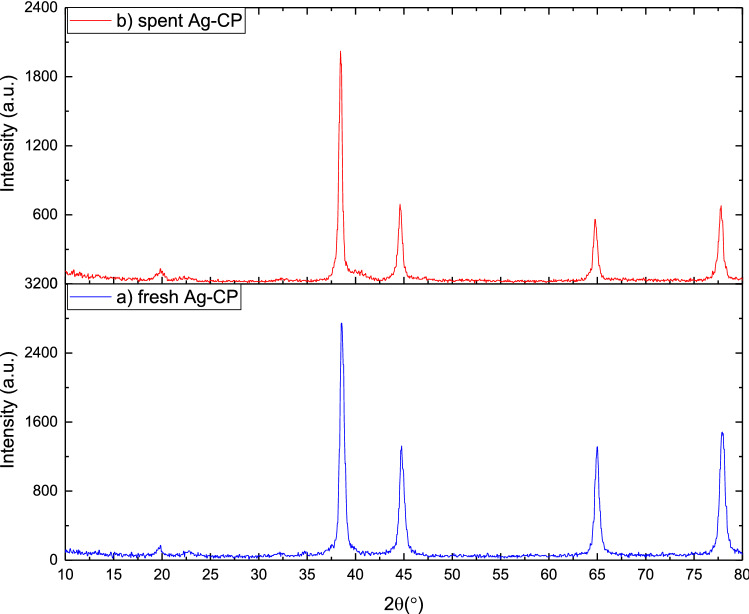
PXRD patterns of the (**a**) fresh Ag–CP and (**b**) spent Ag–CP.

Furthermore, no noticeable changes in crystalline phase can be found from comparison of PXRD of the spent (Fig. 10a) and fresh (Fig. 10b) catalyst. Which shows an excellent physicochemical stability during Hantzsch reaction.

### Comparison of the proposed catalyst with the previously reported catalysts for the Hantzsch condensation

Finally, to evaluate the performance of the present catalytic activity in the unsymmetrical Hantzsch reaction, the current protocol was compared with some of the previously reported catalysts in the synthesis of polyhydroquinolines (Table 3). There is no doubt that all of the listed catalysts in Table 3 can significantly produce the desired product in good to excellent yield. But, it was found out that the Ag–CP as a heterogeneous catalyst can be regarded as superior to almost all of the methods presented in Table 3. These observations may be attributed to the synergistic effect between the Ag and the basic cites in the prepared Ag–CP catalytic system.

**Table 3:** Comparison of the synthesis of polyhydroquinolines in the presence of various catalysts.

Entry	Catalyst	Time (min)	Yield (%)^a^	References
1	FeAl_2_O_4_	180	90	^37^
2	Fe_3_O_4_@D-NH-(CH_2_)_4_-SO_3_H	90	86	^65^
3	Fe_3_O_4_@FSM-16-SO_3_H	25	86	^66^
4	AIL-SCMNPs	15	80	^67^
5	Ag–CP	85	87	This work

^a^Isolated yield.

## Conclusion

In summary, a novel Ag–CP catalytic system was successfully prepared using the reaction of 4,6-diamino-2-mercaptopyrimidine which contains a pyrimidine ring, two amine and one thiol functional groups with strong chelating ability and silver nitrate solutions as commercial available starting materials. Besides, it was characterized by a progressive mode for the preparation. The Ag–CP has an efficient catalytic activity for Hantzsch Synthesis of polyhydroquinolines with various aldehyde-bearing aromatic hydrocarbons in green media and in low reaction times. More importantly, the prepared catalyst affords several advantages such as ease of synthesis, wide range of carriers, ligand-free protocol and easy separation. Simultaneously, the catalyst has remarkable stability and recyclability, and the catalytic activity of the Ag–CP without any significant loss of its activity after four times continuous service.
